# The many roles of an ophthalmic nurse in a tertiary eye institution

**Published:** 2020-12-31

**Authors:** Timothy Adeyemo, Aminatu AbdulRahman, Fatima Kyari

**Affiliations:** 1 Ophthalmic Nurse and Ocular Ultrasonographer: Vitreoretinal Department, National Eye Centre, Kaduna, Nigeria.; 2 Ophthalmologist: National Eye Centre, Kaduna, Nigeria.; 3 Associate Professor: International Centre for Eye Health, London School of Hygiene & Tropical Medicine, UK. Consultant Ophthalmologist: College of Health Sciences, University of Abuja, Nigeria.

**Ophthalmic nurses have to juggle different tasks each day in order to meet the expectations and needs of their patients and colleagues - especially when working in a busy teaching hospital.**

**Figure:**
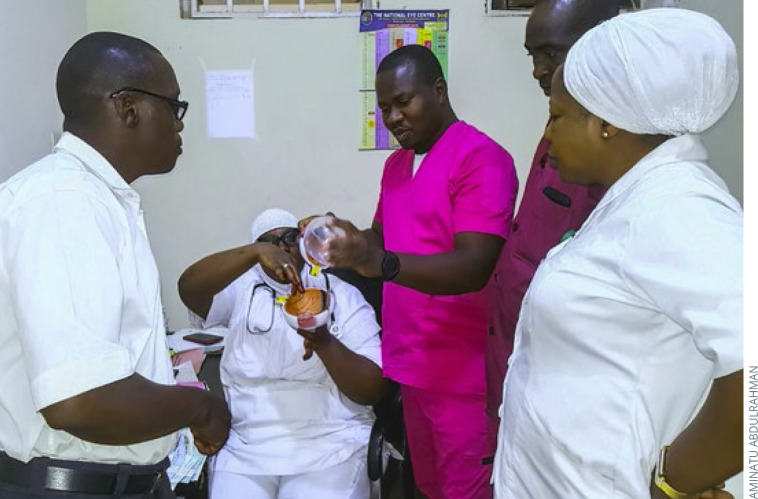
A clinical lecture and demonstration with other colleagues in the vitreoretinal clinic. **NIGERIA**

## Who is the ophthalmic nurse?

Ophthalmic nurses play an important role in global eye health delivery, including eye health promotion; disease prevention, diagnosis and treatment; and low vision and rehabilitation services. In countries where there is a shortage of ophthalmologists, ophthalmic nurses often diagnose and treat patients, referring them where necessary and possible. In this article, we consider the roles of ophthalmic nurses in busy tertiary settings, where they are important members of the eye care team.

**Figure:**
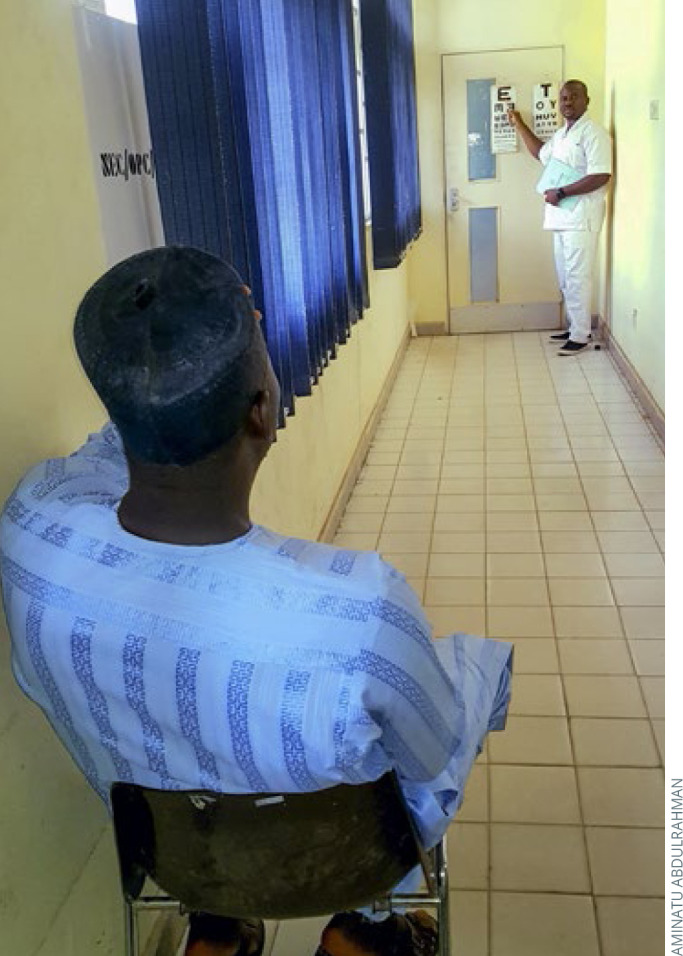
Measuring a patient's visual acuity using the tumbling E chart. **NIGERIA**

## A new era in ophthalmic nursing

Modern ophthalmic nursing is dynamic and constantly evolving to meet the growing demands of patients and the ophthalmic profession. In a tertiary setting, the ophthalmic nurse is often faced with expectations to see more patients, more quickly, embrace new technology and treatments, and use their specialist ophthalmic skills to maintain and enhance the eye health and wellbeing of patients. These additional responsibilities have enabled ophthalmic nurses to broaden their skills and expand their practice in various settings where they are expected to play multiple key roles within the various domains of ophthalmic care.

## Multiple roles

Undertaking multiple roles is influenced by one's skill and experience, and often requires critical thinking skills. Generally, nursing is considered a high-risk, high pressure profession, given the fast-paced working environment and constant need to handle emergencies, especially for those working in low-resource settings where there are high patient-to-nurse ratios. In a tertiary eye hospital, an ophthalmic nurse's role often involves both clinical and administrative duties, carried out in a way that is patient-centered and efficient.

Due to time pressures in a typical eye hospital, and the desire to be accessible to patients,^1^ the ophthalmic nurse with multiple roles is also often under pressure to perform two or more tasks simultaneously. However, such multitasking can increase mistakes and impair one's ability to retain information in working memory.^2^ Therefore, it's important that ophthalmic nurses are permitted and supported to manage their own time and priorities so they can concentrate on, and complete, one task at a time.

In conclusion, we hope this article will contribute to colleagues' and managers' understanding of the complexities of ophthalmic nursing, and that it will help them to recognise the daily achievements of ophthalmic nurses.

Ophthalmic nurses should consider how they can switch between their various roles more efficiently, without becoming distressed or causing harm to patients. For example, they can work on mastering individual tasks and carefully anticipating what may be needed of them next. Ophthalmic nurses can also consider ways of minimising or managing interruptions, especially when performing tasks with a high risk of patient harm if something goes wrong, e.g., when dispensing medication.
