# Supplementary material for: The many roles of an ophthalmic nurse in a tertiary eye institution
Source: Community Eye Health. 2020 Dec 31;33(110):46–7. (PMC8115700)
Supplement: Related texts

# Brief report: From the field

**Published:** 2020

**Timothy Adeyemo** is an ophthalmic nurse with multiple roles in the vitreoretinal clinic at National Eye Centre, Kaduna.

**Figure:** Ophthalmic nurse Timothy Adeyemo performs OCT angiography on a patient in the vitreoretinal clinic. **NIGERIA**

My responsibilities include:

- **Routine examination.** Measuring visual acuity (VA), intraocular pressure (IOP), and vital signs
- **Special clinical investigations.** Carrying out ocular ultrasonography, fundus photography, biometry, ocular coherence tomography (OCT) and OCT A, and fluorescein/indocyanine green angiography
- **Patient counselling.** Counselling and health education
- **Training student nurses.** Organising periodic training or lectures for student nurses and other allied ophthalmic assistants
- **Other.** Providing emergency resuscitation, administration of prescribed medication, assisting the ophthalmologist as necessary, and other administrative duties.

**“I always ensure our patients understand their condition and the management plan.”**

At the vitreoretinal clinic, we attend to an average of 55 patients daily, excluding patients referred for diagnostic investigations. Clinic starts at 7:00am. As patients arrive, the medical health records officer confirms their appointment status. Once at the clinic, we check patients' vital signs, measure their visual acuity and intraocular pressure, and work out their body-mass index (BMI). We document the results in their respective files on the electronic medical record system (EMR) and place patients in a queue so the ophthalmologist can attend to them. The patients are often seen on a first-come, first-served basis, unless they need to be seen urgently, e.g., if the doctor requests that priority be given to older patients or children. The nurses and the community health officers (who work as ophthalmic assistants) work together to ensure a seamless workflow.

**Figure:** Ocular ultrasonography (B-Scan). **NIGERIA**

After the consultation with the ophthalmologist, the patients who require more detailed explanation or counselling are directed to me. Despite the fast pace of work and workload, I always ensure our patients understand the nature of their medical condition and the outlined management plan. I use eye models, illustrated charts, and articles from the *Community Eye Health Journal* to help them understand their condition and management plan. Most of the patients require slow and gentle explanation to ensure they understand. Often, these discussions are continued beyond clinic hours.

At several intervals during the day, I attend to patients referred for different investigations and scans. My ability to switch between roles within a short period of time, while paying attention to every detail, reduces patient waiting time and makes me a valuable member of the team. This is appreciated by the ophthalmologists, who are able to attend to many patients within a short time frame.

My various duties are often interrupted by patients requesting further explanation or clarification of their diagnosis and treatment options. While I consider these interruptions necessary for positive patient outcomes and satisfaction, occasionally they prevent me from completing my tasks within designated timelines, unless I skip my lunch breaks.

## References

[1] S⊘rensenEEBraheL. Interruptions in clinical nursing practice. J Clin Nurs. 2014 May;23(9–10):1274–82. doi: 10.1111/jocn.12329. Epub 2013 Sep 5. PMID: 24003899.2400389910.1111/jocn.12329

[2] SkerrettPJ. Multitasking – a medical and mental hazard. Harvard Health Blog. 2012. **bit.ly/CEHJmultitask**
